# Cellular Distribution of Canonical and Putative Cannabinoid Receptors in Canine Cervical Dorsal Root Ganglia

**DOI:** 10.3389/fvets.2019.00313

**Published:** 2019-09-19

**Authors:** Roberto Chiocchetti, Giorgia Galiazzo, Claudio Tagliavia, Agnese Stanzani, Fiorella Giancola, Marika Menchetti, Gianfranco Militerno, Chiara Bernardini, Monica Forni, Luciana Mandrioli

**Affiliations:** Department of Veterinary Medical Sciences, University of Bologna, Bologna, Italy

**Keywords:** cannabinoid receptor 1, cannabinoid receptor 2, G protein-coupled receptor 55, nuclear peroxisome proliferator-activated receptor alpha, transient receptor potential vanilloid type 1, endocannabinoids, satellite glial cells

## Abstract

Growing evidence indicates cannabinoid receptors as potential therapeutic targets for chronic pain. Consequently, there is an increasing interest in developing cannabinoid receptor agonists for treating human and veterinary pain. To better understand the actions of a drug, it is of paramount importance to know the cellular distribution of its specific receptor(s). The distribution of canonical and putative cannabinoid receptors in the peripheral and central nervous system of dogs is still in its infancy. In order to help fill this anatomical gap, the present *ex vivo* study has been designed to identify the cellular sites of cannabinoid and cannabinoid-related receptors in canine spinal ganglia. In particular, the cellular distribution of the cannabinoid receptors type 1 and 2 (CB_1_ and CB_2_) and putative cannabinoid receptors G protein-coupled receptor 55 (GPR55), nuclear peroxisome proliferator-activated receptor alpha (PPARα), and transient receptor potential vanilloid type 1 (TRPV1) have been immunohistochemically investigated in the C6–C8 cervical ganglia of dogs. About 50% of the neuronal population displayed weak to moderate CB_1_ receptor and TRPV1 immunoreactivity, while all of them were CB_2_-positive and nearly 40% also expressed GPR55 immunolabeling. Schwann cells, blood vessel smooth muscle cells, and pericyte-like cells all expressed CB_2_ receptor immunoreactivity, endothelial cell being also PPARα-positive. All the satellite glial cells (SGCs) displayed bright GPR55 receptor immunoreactivity. In half of the study dogs, SGCs were also PPARα-positive, and limited to older dogs displayed TRPV1 immunoreactivity. The present study may represent a morphological substrate to consider in order to develop therapeutic strategies against chronic pain.

## Introduction

Spinal ganglia, also referred to as dorsal root ganglia (DRG), contain the cell bodies of pseudounipolar primary sensory neurons, which are surrounded by a layer of satellite glial cells (SGCs), also called amphicytes because of their position around each neuron. Chronic pain, both inflammatory and neuropathic, is associated with hyperexcitability of DRG cellular elements and their down-modulation could thereby decrease pain (1). A growing body of literature suggests that cannabinoid receptors play a critical role in nociception through central and peripheral mechanisms (2–8). Recent studies have shed some light on the expression of cannabinoid receptors on neurons and glial cells of the canine nervous system (9–11). In particular, CB_1_ receptor was observed in central nervous system (CNS) neurons (9) and in DRG neurons and glial cells (10), whereas CB_2_ receptor was found in glial cells (astrocytes) of the spinal cord (11).

In addition to the known canonical (i.e., prototypical) cannabinoid receptors CB_1_ and CB_2_, other receptors, such as G protein-coupled receptor 55 (GPR55), nuclear peroxisome proliferator-activated receptor alpha (PPARα), and transient receptor potential vanilloid type 1 (TRPV1) are currently considered putative cannabinoid receptors (12–14).

The anti-nociceptive potential of the endocannabinoid system (15) has prompted the development of therapeutic cannabinoid receptors agonists or medical marjiuana to be used in pets in order to treat chronic pain. The clinical/medical properties of botanical and synthetic cannabinoids in the management of neuropathic pain, allodynia, and chronic non-cancer pain have been recently reviewed (16). Methodological challenges (quali-quantitative variability in cannabinoid content of cannabis plant extracts, inconsistent dosing) as well as acute and chronic impacts on cognition, immune and cardiovascular system are still unsolved issues associated with the therapeutic use of phytocannabinoids (17–20). This is why many research efforts are currently focused on body's own cannabinoids (i.e., endocannabinoids) and related physiological compounds, acting through canonical and putative cannabinoid receptors (15, 21).

Although there is a growing interest in the subject, reliable anatomical studies regarding the cellular distribution of cannabinoid receptors in the canine central and peripheral nervous system (PNS) are still lacking. In order to help filling this anatomical gap, the present *ex vivo* study immunohistochemically investigated the cellular distribution of the cannabinoid and cannabinoid-related receptors CB_1_, CB_2_, GPR55, PPARα, and TRPV1 in cervical DRG of pet dogs.

## Materials and Methods

### Animals

Cervical sensory ganglia and related spinal cord were collected from eight dogs (Table 1). None of them had history of neurological disorders and any gross changes of the spinal cord and vertebral canal. Dogs died spontaneously or were euthanized for human reasons due to different diseases and tissues were collected following owner's permission. According to the Directive 2010/63/EU of the European Parliament and of the Council of 22 September 2010 on the protection of animals used for scientific purposes, the Italian legislation (D. Lgs. n. 26/2014) does not require any approval by competent authorities or ethics committees, because this research did not influence any therapeutic decisions.

**Table 1:** Clinico-pathological data of the dogs included in the present research.

**Dogs**	**Breed**	**Gender**	**Age**	Cause of death^*^
Dog 1	Chihuahua	F	8 months	Head trauma (T)
Dog 2	Great Dane	M	2 years	Peritonitis/ intussusception (V)
Dog 3	Pitbull	M	13 years	Splenic neoplasia, skin neoplasia (N)
Dog 4	Mongrel	M	11 years	Mast cell tumor (N)
Dog 5	Mongrel	F	11 years	Mast cell tumor + Cushing's syndrome (N)
Dog 6	Mongrel	M	14 years	Gastric dilatation-volvulus (V)
Dog 7	Lagotto Romagnolo	F^*^	10 years	Thymoma (N)
Dog 8	Cane Corso Italiano	F	8 years	Gastric tumor (N)

*M, male; F, female; FM, female spayed*.

* *According to the VITAMIND scheme (T, traumatic; V, vascular; N, neoplastic)*.

Since the suppliers of the antibodies employed in the present study state them to rat-specific (CB_2_ and TRPV1) or react with rat tissues (CB_1_, PPARα), rat cervical sensory ganglia were used for comparison purposes (authorization no. 112/2018-PR of 12 February 2018). The distribution of the study receptors in subclasses of rat sensory neurons was out of the scope of the present study, and was not evaluated.

### Tissue Collection

Tissue Samples (C6-C8 DRG) were collected within 1 h from death through a dorsal laminectomy. DRG were localized by counting them from the last cervical spinal nerve (C8) located just cranial to the first rib. C6–C8 cervical DRG were selected for the present study because of technical and pathophysiological implications, i.e., large size, involvement in chronic pain (caused by cervical disk herniation and vertebral column instability), presence of all the subsets of sensory neurons activated by mechanical, thermal and nociceptive inputs from the forelegs. Once removed from the spinal cord, DRG were fixed for 12 h in 4% paraformaldehyde in phosphate buffer (0.1 M, pH 7.2) at 4°C. Tissues were subsequently rinsed overnight in phosphate-buffered saline (PBS; 0.15 M NaCl in 0.01 M sodium phosphate buffer, pH 7.2) and stored at 4°C in PBS containing 30% sucrose and sodium azide (0.1%). The following day, the tissues were transferred to a mixture of PBS−30% sucrose–azide and Optimal Cutting Temperature (OCT) compound (Sakura Finetek Europe, Alphen aan den Rijn, The Netherlands) at a ratio of 1:1 for an additional 24 h before being embedded in 100% OCT in Cryomold® (Sakura Finetek Europe). The sections were prepared by freezing the tissues in isopentane cooled in liquid nitrogen. Serial longitudinal sections (14–16 μm thick) of C6–C8 DRG were cut on a cryostat, and mounted on polylysinated slides.

### Immunofluorescence

Cryosections were hydrated in phosphate-buffered saline (PBS) and processed for immunostaining. To block non-specific bindings, the sections were incubated in a solution containing 20% normal donkey serum (Colorado Serum Co., Denver, CO, USA), 0.5% Triton X-100 (Sigma Aldrich, Milan, Italy, Europe), and bovine serum albumin (1%) in PBS for 1 h at room temperature (RT). The cryosections were incubated overnight in a humid chamber at RT with a cocktail of primary antibodies (Table 2) diluted in 1.8% NaCl in 0.01 M PBS containing 0.1% sodium azide. After washing in PBS (3 × 10 min), the sections were incubated for 1 h at RT in a humid chamber with the secondary antibodies (Table 3) diluted in PBS. Cryosections, were then washed in PBS (3 × 10 min) and mounted in buffered glycerol at pH 8.6.

**Table 2:** Primary antibodies used in the study.

**Primary antibody**	**Host**	**Code**	**Dilution**	**Source**
CB1	Rabbit	Orb10430	1:200	Biorbyt
CB2	Rabbit	ab45942	1:200	Abcam
CD31	Mouse	M0823 Clone JC70A	1:30	Dako
GFAP	Chicken	ab4674	1:800	Abcam
GPR55	Rabbit	NB110-55498	1:200	Novus Biol.
Factor VIII	Rabbit	A0082	1:1,000	Dako
PPARα	Rabbit	NB600-636	1:200	Novus Biol.
Myelin protein zero (P0)	Chicken	ab39375	1:100	Abcam
S100	Rabbit	20311	1:200	Dako
TRPV1(VR1)	Rabbit	ACC-030	1:200	Alomone
VR1 (P-19)	Goat	sc12498	1:50	Santa Cruz

*Primary antibodies Suppliers: Abcam, Cambridge, UK; Alomone, Jerusalem, Israel; Biorbyt Ltd., Cambridge, UK; Dako, Carpinteria, CA, USA; Novus Biologicals, Littleton, CO, USA; Santa Cruz, Biotechnology, CA, USA*.

**Table 3:** Secondary antibodies used in the study.

**Secondary antibody**	**Host**	**Code**	**Dilution**	**Source**
Anti-rabbit 594	Donkey	ab150076	1:100	Abcam
Anti-rabbit 488	Donkey	ab150073	1:800	Abcam
Anti-chicken TRITC	Donkey	703-025-155	1:200	Jackson
Anti-goat 594	Donkey	ab150132	1:600	Abcam
Anti-mouse F(ab')2 fragment TRITC	Goat	ab51379	1:50	Abcam

*Secondary antibodies Suppliers: Abcam, Cambridge, UK; Jackson Immuno Research Laboratories, Inc. Baltimore Pike, PA, USA*.

Cellular nuclei were identified with the DAPI Fluorishield (F6057-20ML, Sigma Aldrich, Milan, Italy, Europe), DRG neurons were identified with the blue fluorescent Nissl staining solution (NeuroTrace®, # N-21479, Molecular Probes, Eugene, OR, USA; dilution 1:200). Satellite glial cells were identified with a polyclonal chicken anti-glial fibrillary acid protein (GFAP) antiserum. Schwann cells were identified with a polyclonal chicken anti-myelin Protein Zero (P0) antiserum. Since CB_2_ receptor may also be expressed by blood vessels (22–24), the endothelial cells were recognized with two different antibodies, i.e., the mouse anti-CD31 antibody (25, 26), and the rabbit anti-Factor VIII-related antigen/von Willebrand factor (27), herein referred to as FVIII-Rag.

In order to determine the proportion of neurons immunoreactive for each of the marker, sections subjected to single immunohistochemistry for cannabinoid receptors were counterstained with blue fluorescent Nissl stain solution (NeuroTrace®, see above) following the manufacturer's instructions. At least one hundred Nissl stained neurons were counted for each marker. Data were collected from preparations obtained from at least three animals (*n* = 3). The percentage of immunopositive neurons was expressed as mean ± standard deviation.

#### Specificity of the Primary Antibodies

The specificity of the anti-cannabinoid receptors CB_1_, CB_2_, and PPARα antibodies in dog tissues has been recently tested by Western blot (Wb) analysis on canine intestinal tissues (24). In the present study we used the antibody anti-human GPR55 (NB110-55498; Novus Bio) which, based on sequence identity (85%), is predicted to cross-react also with canine tissues. However, we tested its specificity on canine tissue by Wb analysis.

To identify TRPV1 immunoreactive neurons, we utilized two different antisera raised in rabbit (Alomone, ACC-030) and goat (Santa Cruz, c12498), directed against two different portions of the rat TRPV1. The immunogen of the rabbit anti-TRPV1 (Alomone) was the peptide [(C)EDAEVFK DSMVPGEK (824–838) of rat TRPV1. The immunogen of the goat anti-VR1 antibody (Santa Cruz) was a synthetic peptide [PHIFTTRSRTRLFGKGDSE(C)] (28–47) from N-terminus of the rat TRPV1. The manufacturer's datasheets for both the anti-TRPV1 antibodies state that the antibodies are specific only for rodents (mouse and rat) and human DRG neurons. The specificity of the goat anti-VR1 antibody has been tested on canine tissues with Wb (48). Thus, we tested the specificity of the two antibodies on rat and canine DRG cryosections beforehand, by using a double-staining protocol. On rat DRG cryosections, the anti-TRPV1 antibody raised in rabbit (Alomone) and the anti-VR1 antibody raised in goat, showed full correspondence within the same neurons, which appeared brightly labeled, providing additional value to the specificity of both the anti-TRPV1 antibodies (data not shown). As observed in porcine DRG (49), only the rabbit anti-TRPV1 antibody identified TRPV1-immunoreactivity in the canine ganglia. However, the specificity of the rabbit anti-TRPV1 antibody was not tested on canine tissues by Wb.

The specificity of the endothelial markers antibodies (anti-CD31 and anti FVIII-Rag) was tested by using a double-staining protocol. Both antibodies recognized the same endothelial cells; however, the antibody anti-CD31 showed a sharper and more delicate immunolabeling of the cells (data not shown). For this reason, the anti-CD31 antibody was used as endothelial marker.

The specificity of the anti-myelin marker protein zero (P0) antiserum was tested by using a double-staining protocol. The anti-P0 antiserum was co-localized with the anti-S100 antiserum; both the myelin markers were co-localized in all the Schwann cells (data not shown).

### Fluorescence Microscopy

Preparations were examined on a Nikon Eclipse Ni microscope equipped with the appropriate filter cubes to distinguish the fluorochromes employed. The images were recorded with a Nikon DS-Qi1Nc digital camera and NIS Elements software BR 4.20.01 (Nikon Instruments Europe BV, Amsterdam, Netherlands). Slight adjustments to contrast and brightness were made using Corel Photo Paint, whereas the figure panels were prepared using Corel Draw (Corel Photo Paint and Corel Draw, Ottawa, ON, Canada).

### Western Blot

**Figure 1:**
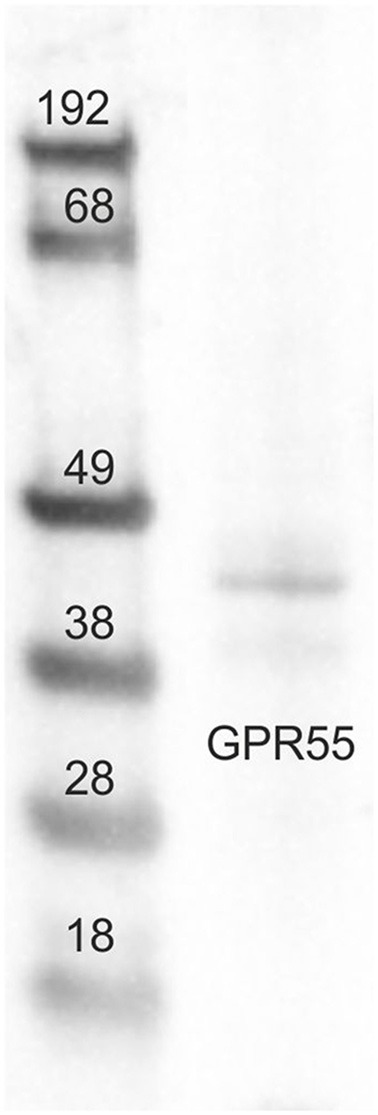
Representative image of Western blots (WB) analysis showing the specificity of the primary antibody rabbit anti-G protein-coupled receptor 55 (GPR55). The antibody revealed a single band of expected molecular weight (~40 kDa). The images of the different immunoblots were slightly adjusted in brightness and contrast to match their backgrounds.

Tissue sample (small intestine/jejunum) was collected, frozen in liquid nitrogen and stored at −80°C until sample processing. Hundred milligram of tissue was homogenized in 1 ml of SDS buffer (Tris-HCl, 62.5 mM; pH 6.8; SDS, 2%; and glycerol, 20%) supplemented with a protease inhibitor cocktail (Sigma-Aldrich, Co, St. Louis, MO, USA). Total protein content was determined by Peterson's Modification of Lowry Method using a Protein Assay Kit. 20 μg of total proteins were separated on NuPage4–12% bis-Tris Gel (Life Technologies Ltd, Paisley, UK) for 30 min at 200 V. The proteins were then electrophoretically transferred onto a nitrocellulose membrane by a semi-dry system (Trans Turbo Blot Bio -Rad). Non-specific binding on nitrocellulose membranes was blocked with 5% milk powder in PBS-T20 (Phosphate Buffer Saline-0.1% Tween-20) for 1 h at room temperature. After blocking treatment, the membrane was incubated overnight at 4°C with the primary antibodies (GPR55 NB110-55498), 1:500 diluted in PBS added with 1.5% of milk. After washes, the blot was incubated with a goat anti rabbit biotin-conjugate antibody (1:50,000 dilution in TBS-T20, 1 h at RT) and then with a 1:1,000 dilution of an anti-biotin horseradish peroxidase (HRP)-linked antibody (40 min at RT). Immunoreactive bands were visualized using chemiluminescent substrate (Clarity Western ECL Substrate Bio Rad), according to the manufacturer's instructions. The intensity of the luminescent signal was acquired by Chemidoc Instrument (Bio Rad) and the apparent molecular weight of the resultant bands was analyzed by Quantity One Software (Bio-Rad). Western blot analysis of GPR55 revealed a single band of expected molecular weight (~40 kDa) (Figure 1).

## Results

### CB_1_ Receptor Immunoreactivity

**Figure 2:**
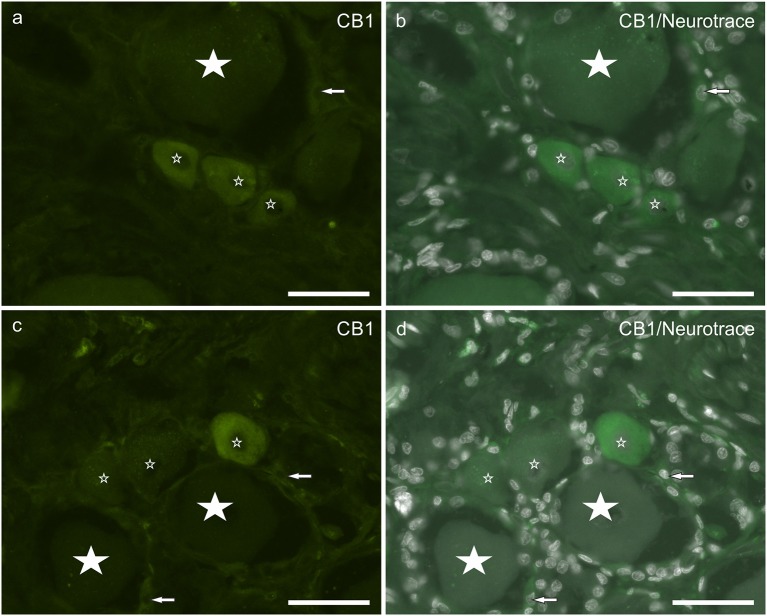
**(a–d)** Photomicrographs of cryosections of canine cervical (C8) dorsal root ganglion showing cannabinoid receptor 1 (CB_1_) immunoreactivity. Small stars indicate small neurons showing CB_1_ receptor weak to moderate immunoreactivity. Large stars indicate CB_1_ receptor negative. Arrows indicate satellite glial cells showing weak CB_1_ receptor immunoreactivity. Bar: a–d = 50 μm.

About half neuronal population (55 ± 6%; 278/507 counted sensory neurons, *n* = 4) displayed weak to moderate cytoplasmic CB_1_ receptor immunoreactivity (Figures 2a–d). CB_1_ receptor immunoreactivity was occasionally observed in SGCs, although it could be confused with background. This finding is partially consistent with observation in the rat DRG, in which neurons and SGCs expressed CB_1_ receptor immunoreactivity also in the nuclei (neurons > SGCs) (Supplementary Figures 1a–c).

### CB_2_ Receptor Immunoreactivity

**Figure 3:**
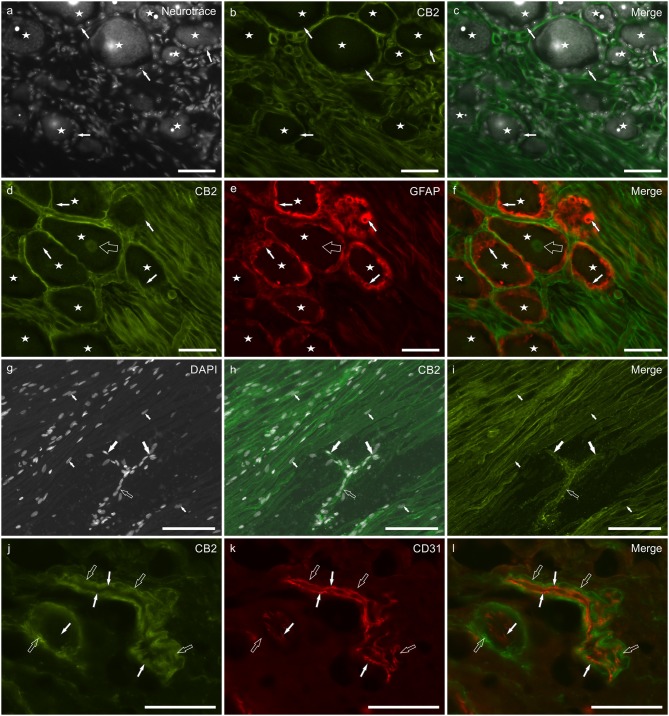
Photomicrographs of cryosections of canine cervical (C8) dorsal root ganglion showing cannabinoid receptor 2- (CB_2_), glial fibrillary acidic protein- (GFAP), and CD31-immunoreactivity. **(a–c)** Stars indicate NeuroTrace labeled **(a)** dorsal root ganglion sensory neurons which were CB_2_ receptor negative **(b)**, as well as the satellite glial cells (white arrows). **(d–f)** Stars indicate sensory neurons encircled by satellite glial cells (white arrows) which were GFAP-immunoreactive **(e)** and CB_2_ receptor negative. CB_2_ receptor immunoreactivity was expressed by Schwann cells and neuronal nuclei (open arrow). **(g–i)** The empty arrow indicates one neuronal axon that bifurcates (T-junction) in its central and peripheral portions (large white arrows). The small arrows indicate the nuclei of Schwann cells. **(j–l)** Open arrows indicate smooth muscle cells (vessel on the left) and pericyte-like cells (elongated and thin blood vessel on the right) showing CB_2_ receptor immunoreactivity **(j)**. White arrows indicate endothelial cells showing CD31 immunoreactivity **(k)**. Bar: **a–f, j–l** = 50 μm; **g–i** = 100 μm.

CB_2_ receptor immunoreactivity was brightly expressed by Schwann cells and cells surrounding blood capillaries (most likely pericytes) (Figures 3a–l), while smooth muscle cells of blood vessels showed moderate CB_2_ receptor immunolabeling (Supplementary Figure 2). SGCs did not display CB_2_ receptor immunolabeling (Figures 3a–f). Faint CB_2_ immunolabeling was expressed by the nuclei of all the DRG neurons (Figures 3d,f). GFAP immunostaining was stronger at the periphery of the ganglia, while CB_2_ receptor immunoreactivity was stronger in the central portion of the ganglia (data not shown). The expression of the CB_2_ receptor on Schwann cells depicted the path of nerve fibers, rolling between neurons before abandoning the ganglion at its central and peripheral pole (Figures 3g–i). In the oldest subjects, the CB_2_ receptor immunolabeling was less intense than in the younger dogs (data not shown). The co-localization of CB2 receptor with the myelin marker P0 showed that both the markers were expressed by all Schwann cells (Supplementary Figures 3a–d). CB_2_ receptor immunoreactivity was brightly expressed by pericyte-like cells (Figures 3j–l). The co-localization study between CB_2_ receptor and the endothelial marker CD31 showed that the endothelium was CB_2_ receptor negative whereas the vascular smooth muscle cells showed faint CB_2_ receptor immunoreactivity (Figures 3j–l). The CB_2_ receptor immunolabeling was also observed within the neuronal nuclei of the rat DRG, whereas Schwann cells and blood vessels were CB_2_ receptor negative (Supplementary Figures 1d–f).

### GPR55 Immunoreactivity

**Figure 4:**
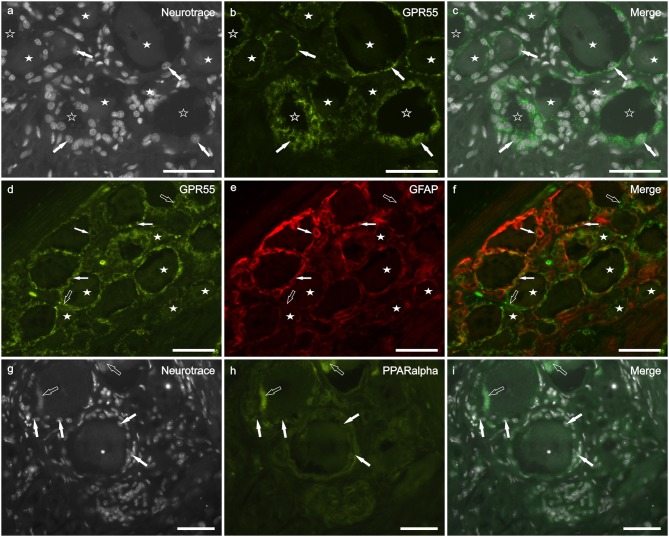
Photomicrographs of cryosections of canine cervical (C8) dorsal root ganglion showing GPR55 **(a–f)** and PPARalpha **(g–i)** immunolabeling. **(a–c)** Arrows indicate the Neurotrace-labeled nuclei of satellite glial cells **(a)** which showed bright GPR55 immunolabelling **(b)**. White stars indicate unlabeled sensory neurons; open stars indicate empty spaces in which sensory neurons were no more evident. **(d–f)** White arrows indicate satellite glial cells which co-expressed bright GPR55- **(d)** and glial fibrillary acidic protein (GFAP) immunoreactivity; open arrows indicate SGCs which were GPR55 immunoreactive and GFAP negative **(e)**. Stars indicate sensory neurons of different dimension, which expressed faint –to-moderate GPR55 immunoreactivity. **(g–i)** White arrows indicate the Neurotrace labeled nuclei of SGCs which showed PPARalpha immunoreactivity **(h)**. Open arrow indicate autofluorescent pigment. Bar: **a–i** = 50 μm.

Bright GPR55 immunoreactivity, with grainy appearance, was expressed by all (GFAP positive and GFAP negative) SGCs (Figures 4a–f). Also a percentage of different size sensory neurons (38 ± 14%; 214/542 cells counted, *n* = 3) showed faint to moderate GPR55 immunolabeling (Figures 4d–f). This finding is consistent with that obtained in neurons and SGCs of the rat DRG (Supplementary Figures 1g–i).

### PPARα Immunoreactivity

PPARα immunoreactivity was expressed by SGCs (Figures 4g–i) and endothelial cells of blood vessels (data not shown). Quite surprisingly, four out of eight dogs did not show PPARα immunoreactivity. In the remainders, all the SGC were PPARα-positive. These data are partially consistent with those obtained in rat DRG, in which also the neuronal cytoplasm showed faint PPARα immunoreactivity (Supplementary Figures 1j–l).

### TRPV1 Immunoreactivity

**Figure 5:**
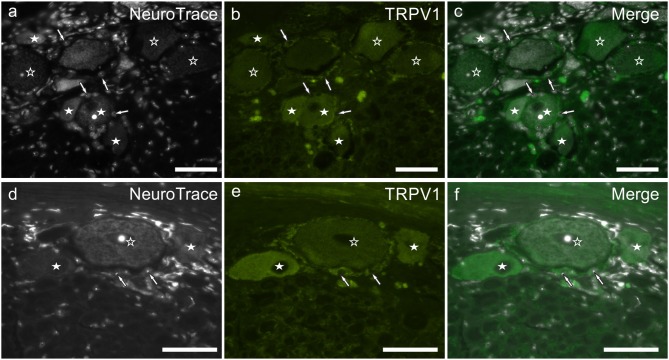
**(a–f)** Photomicrographs of cryosections of the C8 cervical dorsal root ganglia belonging to two aged dogs showing transient receptor potential vanilloid type 1 (TRPV1) immunoreactivity. White stars indicate neurons showing bright TRPV1 immunoreactivity, while open stars indicate larger neurons showing weaker TRPV1 immunoreactivity. Arrows indicate the Neurotrace labeled nuclei of satellite glial cells showing bright TRPV1 immunolabeling **(b,e)**. Bar: **a–f** = 50 μm.

TRPV1 immunoreactivity was unevenly distributed and highly variable within the study cases. In the younger subjects, it was limited to different size neurons (and neuronal processes) while in older dogs, TRPV1 immunolabeling was expressed also by SGCs (Figures 5a–f). In all the subjects, the brightest TRPV1 immunolabeling was displayed by small neurons. The percentage of TRPV1 immunoreactive neurons was 55 ± 11% (563/1,017 cells counted, *n* = 4). In the rat DRG, TRPV1 immunolabeling was expressed only by the cytoplasm of a subset of sensory neurons and nerve fibers (Supplementary Figures 1m–o).

The results of the cellular distribution and intensity of the immunolabeling in the canine DRG are summarized in Table 4.

**Table 4:** Semiquantitative evaluation of the density of CB_1_, CB_2_, GPR55, PPARα, and TRPV1 receptors immunoreactivity in different cellular elements (neurons, satellite glial cells, Schwann cells, blood vessels) of the canine C8 cervical dorsal root ganglia.

	**Canine cervical dorsal root ganglion**				
	**CB1**	**CB2**	**GPR55**	**PPARα**	**TRPV1**
Neurons	C^D^ ++	N^D^ +	C^D^ +	–	C^D^ ++/+++
Satellite glial cells	C^D^ +	–	C^D^ +++	C^D^ ++	C^D^ +++
Schwann cells	–	C^M^ +++	–	–	–
Blood vessels	–	E^D^ +++ SMC^D^ ++	–	E^D^ ++	–

*Immunoreactive cells are graded as: –, negative; +, weakly stained; ++, moderately stained; +++, strongly stained*.

*C, cytoplasmic; D, diffuse labeling; E, endothelium; M, membranes; N, nuclear; SMC, smooth muscle cells*.

## Discussion

The present study showed the expression of canonical and putative cannabinoid receptors in different cellular elements of canine cervical DRG, such as neurons (CB_1_ and GPR55), SGCs (GPR55 and CB_1_), Schwann cells and muscle cells of blood vessels (CB_2_). These findings further substantiate the hypothesis that endogenous ligands, e.g., endocannabinoids and related compounds, may play important roles in modulating the responses associated with hyperexcitability of DRG, such as chronic pain (1). While the role of DRG in pain physiology (i.e., on the crossroads between PNS and CNS) is well-established (50), much less is known about its active involvement in processing chronic pain (1, 51). Given the involvement of the endocannabinoid system in pain modulation (15, 50, 52), our findings may help to shed new light on this challenging issue.

### CB_1_ and CB_2_ Receptors

The expression of CB_1_ receptor in DRG neurons and SGCs is in agreement with previous studies in laboratory rodents (53), humans (54) and dogs (11). However, the neuronal subpopulation expressing CB_1_ receptors (i.e., small sensory neurons) was different from a previous *in situ* hybridization study by Hohmann et al. (55) who found medium-and large-sized cells in rat DRG to predominantly express CB_1_ receptor mRNA. Although, in the present study, the area of DRG neurons was not measured, it is possible to state with some confidence that, in the rat DRG, CB1 receptor immunoreactivity was expressed also by large-sized neurons.

The expression of faint CB_2_ receptor immunolabeling in neurons and its absence in SGCs of canine DRG, partially agrees with previous findings in laboratory rodents, where only very weak immunoflorescence was found in basal conditions (56). Although CB_2_ receptor was considered lacking in neurons and glial cells, recent literature highlights its expression in these cell types (57, 58), even in humans (54) and dogs (11, 59). Similarly to CB_1_ (28), CB_2_ receptor is upregulated in a variety of PNS and CNS diseases and is suggested as a promising pharmacological target in the management of chronic pain and neuroinflammation (29–31, 56). At present we are not able to explain the presence of the CB receptors in neuronal nuclei of canine (CB_2_ receptor) and rat (CB_1_ and CB_2_ receptors) DRG. The study on the subcellular distribution and function of cannabinoid receptors is still expanding. The nuclear envelope, which is a part of the endoplasmic reticulum, may be one of the sources of nuclear Ca^2+^; Curry et al. (60) identified the expression of CB_1_ and CB_2_ receptors on the nuclear membrane of cardiac muscle cells and demonstrated that these receptors, when activated by anandamide, can (negatively) modulate nuclear Ca^2+^ release and, very likely, gene transcription.

To the best of our knowledge, this is the first time that CB_2_ receptor immunoreactivity in Schwann cells has been reported. Up to now, endocannabinoid receptor immunolabeling of Schwann cells was limited to CB_1_, which was shown in about 100% of this cell type in the canine sciatic nerve (10). Besides forming the myelin sheath, Schwann cells orchestrate much of the regenerative response that occurs after nerve injury in order to restore nerve function (32). The expression of CB_1_ (10) and CB_2_ receptors (present study) in Schwann cells could thus support the neuroprotective and/or neuroreparative role suggested for cannabinoids and related compounds in the PNS (33, 56).

The presence of thin interneuronal GFAP-negative cellular processes expressing CB_2_ receptor-immunoreactivity is at present not easy to interpret. These CB_2_ receptor immunoreactive slender evaginations might belong to GFAP-negative SGCs (34) or to a different type of DRG glial cells, i.e., pericyte-like satellite cells (35, 36). Also the presence of different cell types with elongated cellular processes immunoreactive for CB_2_ receptor, such as fibroblasts and histiocytes (34, 36), cannot be excluded.

Some considerations are needed when dealing with DRG blood vessels. First, little information is available and it mainly refers to laboratory rodents. Second, blood-nerve barrier is lacking in intact DRG (37) and fenestrations together with open intercellular junctions characterize ganglionic vessels (38, 39). Although the sheath of SGCs is considered to control the traffic of substances from blood to ganglionic neurons—thus functionally substituting for the vascular barrier (40)—circulating signaling molecules are allowed to diffuse into the microenvironment of DRG. This was recently confirmed by Svíženská et al. (56), who demonstrated that sciatic nerve injury induces bilateral increase of CB_2_ receptor (both protein and mRNA) in lumbar L4–L5 as well as cervical C7–C8 DRG.

In the present study we detected CD31 and FVIII-RAg immunoreactivity in a small proportion of DRG vessels, mostly confined to the periphery of the ganglion rather than among sensory neurons. The finding is quite unexpected, since the endothelial marker CD31 allowed to trace an extensive network of blood vessels in the mouse L4 DRG, that was found to encapsulate and encircle sensory neurons (41). The paucity of vascularization of canine DRG did not seem to depend on methodological issues since the antibody anti-CD31 was recently found to perfectly label the endothelium of canine blood vessels, at least in the intestinal mucosa (24).

In the present study CB_2_ receptor immunoreactivity was limited to smooth muscle cells of blood vessels, being absent from CD31-positive endothelium, differently from what observed in canine intestinal (24) and skin blood vessels (42), or human brain endothelium (43). One possible explanation for this discrepancy might be the well-known regional distribution of the cannabinoid receptors in blood vessels (44). Indeed, CB_2_ receptor immunoreactivity of vascular smooth vessels was recently detected in bovine pancreas (45) and mice skin (46). Endocannabinoids exert a prohomeostatic function on vascular biology through complex mechanisms often involving canonical as well as putative cannabinoid receptors [e.g., TRPV1 and GPR55 (47)]. In particular, vasodilating effect occurs at different cellular site, i.e., nerves, endothelial cells, vascular smooth muscle cells, perycites (61), employing different receptors and leading to nitric oxide release (47).

### GPR55

The GPR55 represents a novel target for various cannabinoids (62). Strong expression of GPR55 immunoreactivity in of different size neurons and SGCs was found in the present study. GPR55 immunoreactivity was expressed also by GFAP negative SGCs; a recent study showed that GFAP recognizes up to 89% of all SGCs of the canine DRG (34). This finding indicates that GPR55 might be utilized as canine SGCs marker. In the present study, a similar pattern of GPR55 immunoreactivity has been observed also in the neurons and SGCc of rat DRG. This is a relatively new finding, since up to now GRP55 immunoreactivity has been detected only in the neuronal component of DRG (63). Consistently, the GPR55 immunoreactivity in medium- and large-sized DRG neurons as detected here agrees with the finding of Lauckner et al. (63), who observed strong GPR55 signal in mice DRG large neurons. Interestingly, large sensory neurons may mediate inflammatory and neuropathic pain hypersensitivity by switching their phenotype and expressing the nociceptive neurotransmitter Substance P (64, 65). It is noteworthy to recall that some phytocannabinoids, e.g., Δ^9^-tetrahydrocannabinol (THC), cannabidiol, synthetic cannabinoids (AM251 and O-1602), as well as palmitoylethanolamide (PEA) have been described as GPR55 ligands (7, 66).

Although further functional investigations are necessary, GPR55 immunoreactivity in both SGCs and neurons as detected in the present study likely may suggest a relevant role of this receptor in neuron-SGCs crosstalk, which is currently considered a critical component of neuroinflammatory changes eventually leading to chronic pain (67–70).

### PPARα

The PPARα is a ligand-activated transcription factor belonging to the superfamily of nuclear hormone receptors. By modulating gene expression, it plays key roles in maintaining glucose and lipid homeostasis and inhibiting inflammation (71). The PPARα activation has also been shown to induce rapid, cellular changes without requiring transcription (72). In the present study PPARα immunoreactivity has been detected in the canine SGCs and endothelial cells. In the comparative study on rat DRG, we observed bright PPARα immunoreactive SGCs, whereas neurons wear faintly immunolabeled. These findings are in line with previous data on the expression of PPARα in mice DRG (73–75) and canine gastrointestinal tract (24). The ganglia of four out of eight dogs did not show PPARα immunoreactivity. At present we do not have any clear explanation for this discrepancy. No apparent correlation with any particular factor (e.g., age or cause of death) was found. Nonetheless, we cannot exclude that it was due to an undetected subclinical state, given that metabolic disorder, for example, is associated with significantly decreased spinal PPARα expression (76).

### TRPV1

The TRPV1 is a ligand-gated non-selective cation channel usually expressed by peptidergic nociceptors of rodents (77, 78) and large mammals (49) as well as non-peptidergic nociceptors (79, 80). The TRPV1 is activated by heat (>43°C), low pH and capsaicin (81) and desensitized by endocannabinoids (82, 83).

In accordance with previous studies in rodent and human DRG (54, 81, 84, 85) we have observed diffuse TRPV1 immunoreactivity in neurons of canine DRG, with the brightest immunolabeling being displayed by small size neurons. This latter finding agreed with the study of Binzen et al. (86), who found TRPV1 to be mainly expressed in small-sized neurons of rat DRG, the vast majority of which co-expressed CB_1_ receptors. Our comparative study on rat DRG confirmed that the brightest TRPV1 immunoreactivity was mainly expressed by small neurons. Moreover, SGCs from two old dogs were also brightly immunolabeled, in accordance with TRPV1 expression by DRG glial cells (87).

To the best of our knowledge no information is yet available about the influence of age on neuronal and/or glial expression of TRPV1, however one could tentatively speculate that aging itself has an impact on pain pathophysiology through changes in the pain involved receptor TRPV1. Actually, increased expression of TRPV1 was recently observed in rat DRG after neuropathic pain induction (88). Marrone et al. (89) reported TRPV1 immunoreactivity in microglial cells rather than neurons of the mice brain areas. Moreover, they showed that in mice suffering from neuropathic pain, TRPV1 was also functionally expressed in cortical neurons. Together with the present morphological data, the findings by Marrone et al. (89) indicate that TRPV1 might be a key player of glia-neuron communication.

Recent studies have shown that TRPV1 is desensitized by a number of cannabinoids, including THC, cannabinol, synthetic cannabinoid WIN 55,212-2, AEA, rimonabant (7) as well as PEA (83, 90–92). This ability is very important as TRPV1 channel desensitization is considered to be responsible for analgesic and anti-inflammatory effects (89).

A limitation of the study is the lack of unquestionable specificity test of the employed TRPV1 antibody in dog tissue. The TRPV1 has been cloned and functionally characterized from different species, including dogs. Peptide alignment of the dog TRPV1 ortholog with other species of the TRPV1 family revealed a high degree of sequence homology (human, 89.1%; rat, 87.5%; mouse, 83.3%) (93). Actually, the antibody performs well in an optimized IHC assay, binding the indicated target, not only in dog tissue (TRPV1 immunolabeled SGCs were observed also in cat and horse cervical DRG, while in small rodents and guinea-pig the TRPV1 immunoreactivity was always limited to DRG neurons—RC personal observation). Thus, since the dog was proposed as a good model for studying the role of TRPV1 in inflammatory diseases and nociception and the effects of TRPV1 antagonists in humans (93), additional molecular analysis, such as knockout cell lines and Western blot (assuming the IHC-based antibody also works in Western blots), might be necessary to strength the results of TRPV1 immunolabeling, and to increase confidence for the validity in the dog.

## Conclusion

The present study highlighted the expression of canonical and putative cannabinoid receptors on different DRG cell types, in particular neurons and glial cells (SGCs and Schwann cells). Given the key role of DRG elements and cannabinoid receptors in the pathophysiology of chronic pain, targeting and modulating these receptors, possibly through a multifaceted approach, may become a novel way to manage pain in veterinary patients.

## Data Availability

All datasets generated for this study are included in the manuscript/Supplementary Files.

## Author Contributions

RC, LM, and GM: study concept and design. Western blot analysis was carried out by CB and MF. The immunohistochemical experiments were carried out by FG, GG, AS, MM, and CT. RC and GG: acquisition of data. All authors interpreted the data. RC: drafting of the manuscript and study supervision. All authors contributed to revision of the article for critical intellectual content and have approved the final version.

### Conflict of Interest Statement

The authors declare that the research was conducted in the absence of any commercial or financial relationships that could be construed as a potential conflict of interest.
