# Corrigendum: Cellular Distribution of Canonical and Putative Cannabinoid Receptors in Canine Cervical Dorsal Root Ganglia

**DOI:** 10.3389/fvets.2019.00377

**Published:** 2019-10-31

**Authors:** Roberto Chiocchetti, Giorgia Galiazzo, Claudio Tagliavia, Agnese Stanzani, Fiorella Giancola, Marika Menchetti, Gianfranco Militerno, Chiara Bernardini, Monica Forni, Luciana Mandrioli

**Affiliations:** Department of Veterinary Medical Sciences, University of Bologna, Bologna, Italy

**Keywords:** cannabinoid receptor 1, cannabinoid receptor 2, G protein-coupled receptor 55, nuclear peroxisome proliferator-activated receptor alpha, transient receptor potential vanilloid type 1, endocannabinoids, satellite glial cells

In the original article, there was a mistake in the legend for **Figure 3** as published. The legend of Figure 3 (g-l) is incorrect. The correct legend appears below.

**FIGURE 3** | Photomicrographs of cryosections of canine cervical (C8) dorsal root ganglion showing cannabinoid receptor 2- (CB_2_), glial fibrillary acidic protein- (GFAP), and CD31-immunoreactivity. **(a–c)** Stars indicate NeuroTrace labeled **(a)** dorsal root ganglion sensory neurons which were CB_2_ receptor negative **(b)**, as well as the satellite glial cells (white arrows). **(d–f)** Stars indicate sensory neurons encircled by satellite glial cells (white arrows) which were GFAP-immunoreactive **(e)** and CB_2_ receptor negative. CB_2_ receptor immunoreactivity was expressed by Schwann cells and neuronal nuclei (open arrow). **(g–i)** The empty arrow indicates one neuronal axon that bifurcates (T-junction) in its central and peripheral portions (large white arrows). The small arrows indicate the nuclei of Schwann cells. **(j–l)** Open arrows indicate smooth muscle cells (vessel on the left) and pericyte-like cells (elongated and thin blood vessel on the right) showing CB_2_ receptor immunoreactivity **(j)**. White arrows indicate endothelial cells showing CD31 immunoreactivity **(k)**. Bar: **a–f, j–l** = 50 μm; **g–i** = 100 μm.

The authors apologize for this error and state that this does not change the scientific conclusions of the article in any way. The original article has been updated.

