# Supplementary material for: Cellular Distribution of Canonical and Putative Cannabinoid Receptors in Canine Cervical Dorsal Root Ganglia
Source: Front Vet Sci. 2019 Sep 19;6:313. doi: 10.3389/fvets.2019.00313 (PMC6761858; doi:10.3389/fvets.2019.00313)
Supplement: Supplementary file 1 [file Image_1.pdf]

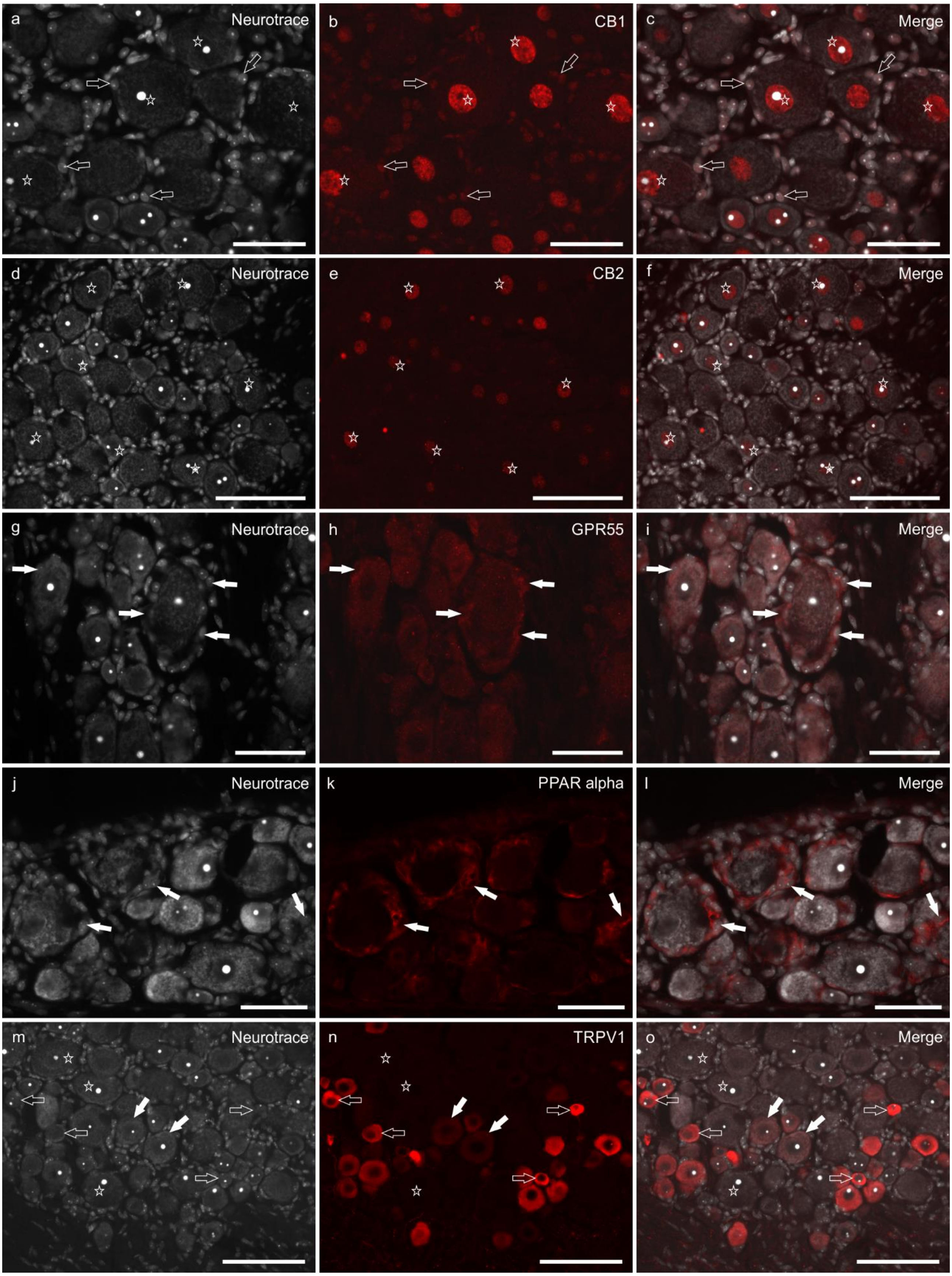

Supplementary Figure 1. Photomicrographs of cryosections of rat cervical (C8) dorsal root ganglion showing CB1 (a-c), CB2 (d-f), GPR55 (g-i), PPARalpha (j-l), and TRPV1 (m-o) immunolabeling. a-c) CB1 immunoreactivity was brightly expressed by the nuclei of sensory neurons (stars), whereas the nuclei of satellite glial cells (arrows) showed weaker CB1 immunolabeling. The neuronal

cytoplasm showed, on the contrary, weak or undetectable CB1 receptor immunoreactivity d-f) Stars indicate some of the nuclei of the sensory neurons expressing CB2 immunoreactivity. g-i) Sensory neurons expressing weak to moderate GPR55 immunoreactivity; arrows indicate the nuclei of some satellite glial cells which expressed brighter GPR55 immunoreactivity. j-l) Arrows indicate the nuclei of SGCs expressing bright PPARalpha immunoreactivity. m-o) Arrows indicate sensory neurons expressing bright TRPV1 immunoreactivity. Larger neurons were TRPV1 negative (stars) or showed weaker TRPV1 immunolabeling (white stars).

Bar: a-c, g-l = 50 µm; d-f, m-o = 100 µm.
