# Supplementary material for: Cellular Distribution of Canonical and Putative Cannabinoid Receptors in Canine Cervical Dorsal Root Ganglia
Source: Front Vet Sci. 2019 Sep 19;6:313. doi: 10.3389/fvets.2019.00313 (PMC6761858; doi:10.3389/fvets.2019.00313)
Supplement: Supplementary file 2 [file Image_2.pdf]

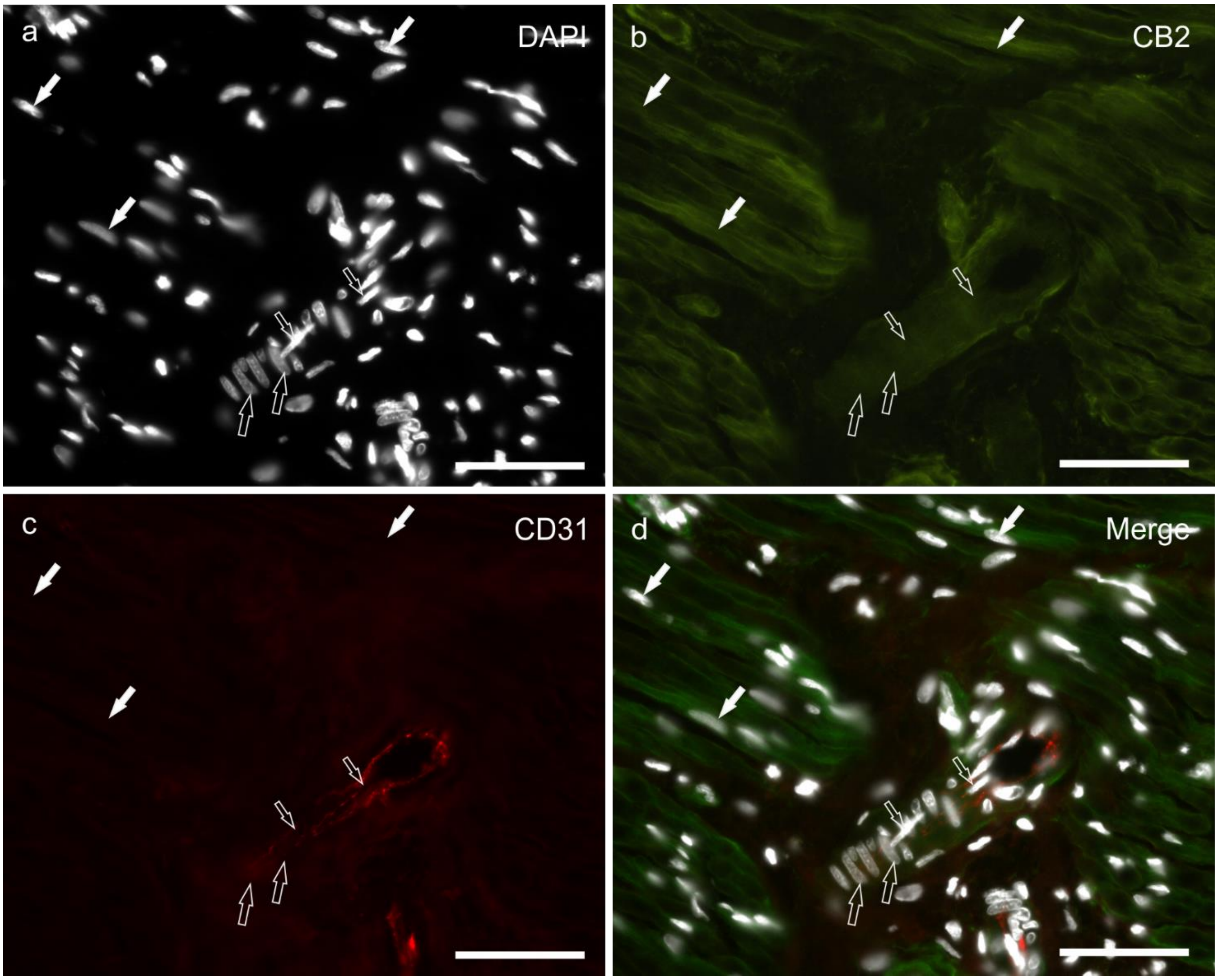

Supplementary Fig. 2. a-d) Photomicrographs of cryosections of canine cervical (C8) dorsal root ganglion (DRG) showing cannabinoid receptor 2- (CB2) and endothelial marker CD31 immunoreactivity. Large and empty arrows indicate the DAPI labelled nuclei of vascular smooth muscle cells (a), which showed weak CB2 receptor immunoreactivity (b). The small empty arrows indicate the nuclei of CD31 immunoreactive endothelial cells (c). White arrows indicate the nuclei of CB2 receptor immunoreactive Schwann cells (b).

Bar: a-d= 50 µm
