# Supplementary material for: Cellular Distribution of Canonical and Putative Cannabinoid Receptors in Canine Cervical Dorsal Root Ganglia
Source: Front Vet Sci. 2019 Sep 19;6:313. doi: 10.3389/fvets.2019.00313 (PMC6761858; doi:10.3389/fvets.2019.00313)
Supplement: Supplementary file 3 [file Image_3.pdf]

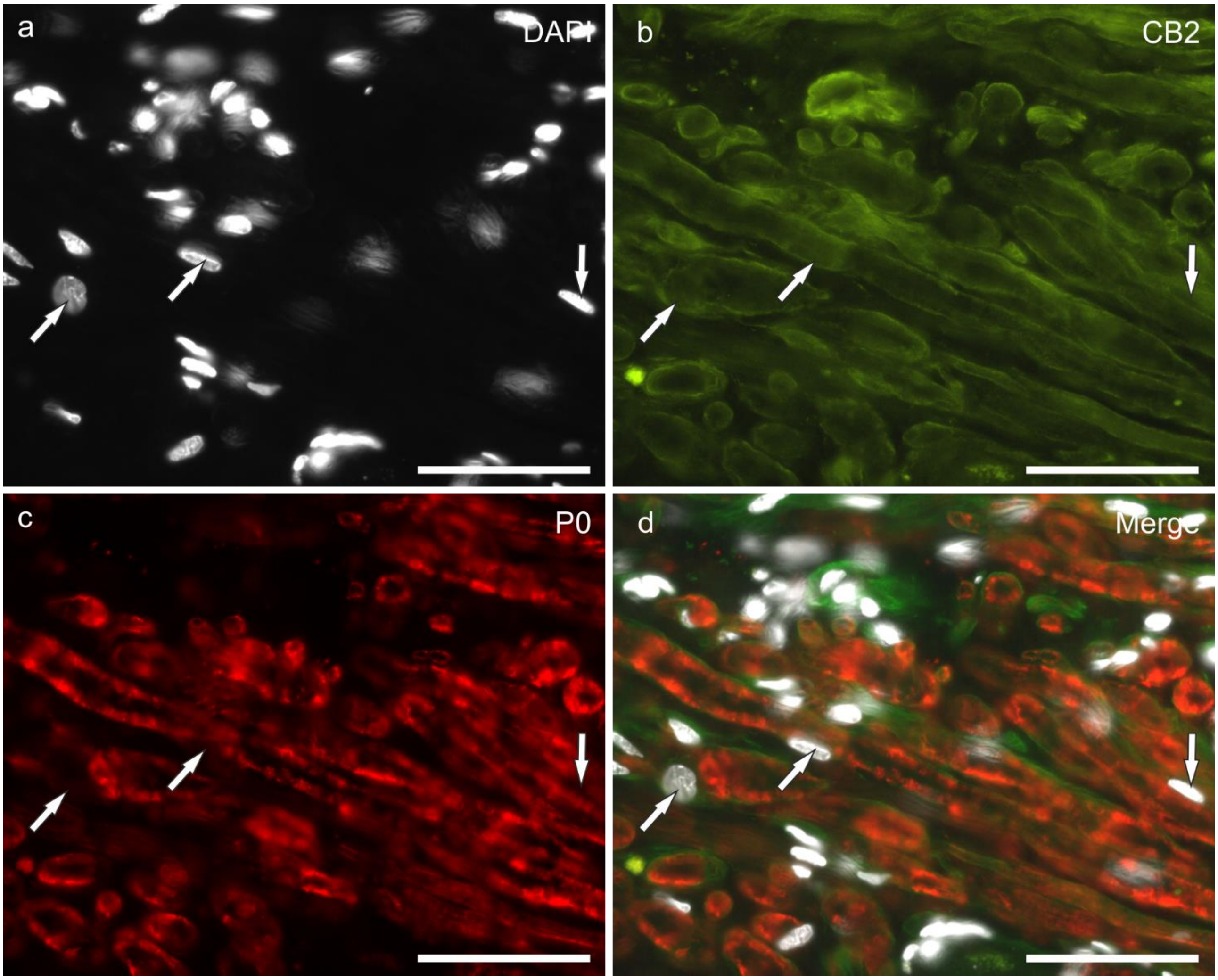

Supplementary Fig. 3. a-d) Photomicrographs of cryosections of canine cervical (C8) dorsal root ganglion (DRG) showing cannabinoid receptor 2- (CB2) and myelin protein zero (P0) immunoreactivity. Arrows indicate the nuclei of Schwann cells showing co-localization between CB2 receptor and P0 immunoreactivity.

Bar: a-d= 50 µm
